# After Bone Marrow Transplantation, the Cell-Intrinsic Th2 Pathway Promotes Recipient T Lymphocyte Survival and Regulates Graft-versus-Host Disease

**DOI:** 10.4049/immunohorizons.2300021

**Published:** 2023-06-09

**Authors:** Jamie Truscott, Xiaoqun Guan, Hope Fury, Tyler Atagozli, Ahmed Metwali, Weiren Liu, Yue Li, Robert W. Li, David E. Elliott, Bruce R. Blazar, M. Nedim Ince

**Affiliations:** * Department of Pediatrics, Carver College of Medicine, University of Iowa, Iowa City, IA; † Department of Internal Medicine, Carver College of Medicine, University of Iowa, Iowa City, IA; ‡ Veterans Administration Medical Center, Iowa City, IA; § Animal Parasitic Diseases Laboratory, United States Department of Agriculture, Agricultural Research Service, Beltsville, MD; ¶ Holden Comprehensive Cancer Center, Carver College of Medicine, University of Iowa, Iowa City, IA; ‖ Division of Blood and Marrow Transplantation & Cellular Therapy, Department of Pediatrics, University of Minnesota, Minneapolis, MN

## Abstract

Recipient T cells can aggravate or regulate lethal and devastating graft-versus-host disease (GVHD) after bone marrow transplantation (BMT). In this context, we have shown before that intestinal immune conditioning with helminths is associated with survival of recipient T cells and Th2 pathway–dependent regulation of GVHD. We investigated the mechanism of survival of recipient T cells and their contribution to GVHD pathogenesis in this helminth infection and BMT model after myeloablative preparation with total body irradiation in mice. Our results indicate that the helminth-induced Th2 pathway directly promotes the survival of recipient T cells after total body irradiation. Th2 cells also directly stimulate recipient T cells to produce TGF-β, which is required to regulate donor T cell–mediated immune attack of GVHD and can thereby contribute to recipient T cell survival after BMT. Moreover, we show that recipient T cells, conditioned to produce Th2 cytokines and TGF-β after helminth infection, are fundamentally necessary for GVHD regulation. Taken together, reprogrammed or immune-conditioned recipient T cells after helminth infection are crucial elements of Th2- and TGF-β–dependent regulation of GVHD after BMT, and their survival is dependent on cell-intrinsic Th2 signaling.

## Introduction

Graft-versus-host disease (GVHD) is a lethal and devastating complication of bone marrow (BM) transplantation (BMT) or hematopoietic cell transplantation (HCT). It affects approximately half of BMT/HCT recipients (1). The organ damage caused by GVHD is a result of donor T cell–mediated inflammation, with other immune cell populations also contributing to tissue injury. GVHD-related end-organ toxicity by donor cells is triggered by pretransplant preparation of recipients with radiation and/or chemotherapy (2, 3). These preparation regimens are primarily used to open space for donor hematopoietic stem cell engraftment and, secondarily, when BMT or HCT is performed to treat a hematological malignancy, to kill residual tumor cells (4). This conditioning varies in intensity, level of myeloablation, and immune suppression. More intense conditioning, including total body irradiation (TBI), results in complete myeloablation and profound immune suppression. Using the right dose of myeloablative or nonmyeloablative preparation in combination with other therapeutic modalities (such as mAbs) has been actively investigated to reprogram donor and the surviving recipient immune cell populations after BMT and to promote transplantation tolerance (5).

Recipient cell groups that survive the pretransplant preparation play critical roles in GVHD pathogenesis and activation of donor T lymphocytes, especially before donor cell expansions populate the tissues of recipients. For example, a recipient’s professional APCs such as dendritic cells (6, 7) or epithelial cells (8) activate donor T lymphocytes by presenting pleomorphic transplantation Ags, present in the recipient but absent in the donor. Surviving recipient T cells can also play a role in initiation or maintenance of tissue inflammation, although recipient T cell subsets can as well regulate transplant immunity (9–13). In this context, recipient T cell–generated modulatory cytokines have been shown to dampen donor T cell activation and the subsequent cascade of events that results in GVHD (14, 15).

Several studies including our previous work have demonstrated that Th2-specific IL-4 or immune regulatory TGF-β belongs to this group of modulatory cytokines (14–18). These cytokines regulate GVHD and promote the survival of BMT recipients. In our previous experiments, we used a myeloablative BMT model after preparation with TBI and activated the Th2 pathway by colonizing the gut of BMT mice with helminths. As a model of helminth infection to condition intestinal immunity, we used the nematode *Heligmosomoides polygyrus bakeri* (*Hpb*). Helminth-induced activation of the Th2 pathway resulted in augmented IL-4 and IL-4–dependent TGF-β production by recipient and donor T cells, and helminth colonization regulated GVHD in a TGF-β–dependent manner (14, 17, 18).

Recipient T cells can also cause GVHD-associated tissue damage independent of donor T cells (13, 19) and thereby contribute to disease pathogenesis instead of regulating graft-versus-host reactivity. Our previous studies demonstrated increased survival of recipient T cells in helminth-infected BMT recipients (18). The mechanism of recipient T cell survival after TBI or BMT in helminth-infected mice and its biological significance are unknown.

In the current study, we investigated the mechanism of recipient T cell survival after BMT in helminth-infected mice and characterized how the presence of recipient T lymphocytes alters helminthic immune regulation. Our results show that stimulation of the Th2 pathway of mice after helminth infection directly augments TGF-β production by recipient T cells and directly promotes T lymphocyte survival after TBI. Moreover, we demonstrate that recipient T cells, reprogrammed to produce Th2 cytokines and TGF-β after helminth infection, are critical for GVHD regulation.

## Materials and Methods

### Mice and *H. polygyrus bakeri* administration

Wild-type (WT) C57BL/6 (MHC type: H2^b^), WT BALB/c (H2^d^), IL-4^−/−^ (H2^d^), Il4Rα^−/−^ (H2^d^), STAT6^−/−^(H2^d^), and RAG^−/−^ (H2^d^) mice, as well as mice with a T cell–specific defect in TGF-β signaling due to overexpression of a truncated TGF-β receptor II (Cd4-TGFBR2; also called TGF-β receptor II dominant negative [TGF-βRII DN]) (H2^b^), were received from The Jackson Laboratory (Bar Harbor, ME) and maintained at the pathogen-free facilities of the University of Iowa. Mice with a T cell–specific deficiency for the Il4Rα gene were generated by crossing iLck Cre (H2^d^) to Il4Rα^flox/flox^ (H2^d^) mice (20), and both strains were a gift of Dr. Frank Brombacher (Cape Town, South Africa). Five- to 6-wk-old WT BALB/c, IL-4^−/−^, Il4Rα^−/−^, STAT6^−/−^, RAG^−/−^, and iLck Cre-Il4Rα^flox/flox^ (conditional knockout [CKO]) (all H2^d^) mice were inoculated with 150 *Hpb* third-stage larvae via oral gavage. We used a modified Baermann method to obtain and enrich for infective *Hpb* third-stage larvae (original specimens archived at the U.S. National Helminthological Collection, no. 81930) from stool of helminth (*Hpb*)-infected mice (21). Initially, some *Hpb* larvae were also provided by Dr. Joseph F. Urban (U.S. Department of Agriculture, Beltsville, MD). All procedures regarding parasite passage and maintenance were strictly carried out according to the animal use protocol approved by the U.S. Department of Agriculture Beltsville Area Institutional Animal Care Committee (protocol no. 21-019). Infective larvae were stored at 4°C until used. All mice were maintained and used in accordance with the guidelines of the University of Iowa Animal Care and Use Committee.

### Cell purification for GVHD induction

Donor (graft) BM cells were obtained from the tibias and femurs of uninfected 5- to 8-wk-old WT C57BL/6 mice. Samples were depleted of T cells (T cell-depleted [TCD]) using mouse pan T cell beads (Dynabeads mouse pan T [Thy 1.2], Invitrogen) according to the manufacturer’s instructions. Samples of spleen cells from uninfected 5- to 8-wk-old C57BL/6 mice were enriched for donor T lymphocytes (CD3^+^) using a T cell isolation kit (Dynabeads untouched mouse T cells, Invitrogen).

### TBI and GVHD induction

Our studies used an acute lethal GVHD model with MHC class I/II (H2^b^→H2^d^) mismatch. H2^d^ recipient strains (see above) carry the MHC class I genes H2K^d^, H2D^d^, and H2L^d^, besides carrying the MHC class II genes I-A^d^ and I-E^d^. In contrast, H2^b^ donor strains carry the MHC class I genes H2K^b^ and H2D^b^, besides carrying the MHC class II gene I-A^b^ (the H2^b^ donor strains we used do not carry H2L or I-E genes). The MHC class I/II mismatch between the donor and recipient causes acute lethal GVHD after TBI, although donor and recipient strains share MHC class Ib genes. Three-week-old *Hbp*-infected and uninfected WT BALB/c, IL-4^−/−^, Il4Rα^−/−^, RAG^−/−^, CKO, and STAT6^−/−^ recipients (all H2^d^) were subjected TBI using a [^137^Cs] source (total of 850 cGy given in two doses given 4 h apart), after which 10 × 10^6^ TCD-BM cells with or without 1.5 × 10^6^ purified splenic T lymphocytes from uninfected C57BL/6 WT (H2^b^) donors were administered i.v. via retroorbital injection. In some experiments, 1.5 × 10^6^ purified splenic T lymphocytes from uninfected Cd4-TGFBR2 mice were administered with 10 × 10^6^ TCD-BM cells from C57BL/6 WT mice as donor cells. Mice were monitored daily for survival for up to 80 d. Disease severity was scored based on animal weight, posture, activity, fur texture, and skin integrity (22–24). In parallel experiments, uninfected and *Hpb*-infected mice were sacrificed 6 d after BMT and subjected to analysis of cell composition by flow cytometry and grading of inflammation based on histopathology.

For the analysis of T cell survival after TBI, *Hpb*-infected or uninfected WT BALB/c, IL-4^−/−^, Il4Rα^−/−^, STAT6^−/−^, and CKO (H2^d^) mice (all H2^d^) underwent TBI using a [^137^Cs] source (total of 850 cGy split into two doses given 4 h apart). Six days later, the cell compositions of the spleen and mesenteric lymph nodes (MLNs) were analyzed by flow cytometry.

### Flow cytometry

Six days after BMT, uninfected and *Hpb*-infected mice were sacrificed, and the spleen and MLNs were isolated for analysis of cell composition. For surface staining, 5 × 10^6^ cells were suspended in PBS with 2% FCS, and Fc receptors were blocked with 2.4G2 mAb (clone 93, BioLegend). The following Abs were used for surface staining: anti-CD4 FITC/PE/PE-Cy7/allophycocyanin (clone GK1.5), anti-CD3 PE-Cy7/PerCP (clone 17A2), and anti-H2b BV421/allophycocyanin/PE (clone AF6-88.5, eBioscience). Intracellular staining for Foxp3 was performed using anti-Foxp3 (PE/allophycocyanin/PE-Cy7) (clone FJK-16S, eBioscience) in accordance with the manufacturer’s instructions.

### Cell purification for in vitro cultures

CD4^+^ T cells were purified from MLNs of *Hpb*-infected and uninfected WT BALB/c and Il4Rα^−/−^ mice using a CD4 T cell isolation kit (Miltenyi Biotec); this resulted in >98% enrichment for CD4 T cells (data not shown). Cells were stimulated with plate-bound anti-CD3 (clone 145-2C11, eBioscience) and soluble anti-CD28 (clone 37.51, eBioscience) (each at 1 μg/ml). TGF-β content was analyzed in acidified and realkalinized supernatants of 72-h cultures in cell culture medium with 1% FCS and 1 mg/ml BSA, using Ab pairs from R&D Systems, and according to the manufacturer’s instructions (14, 17).

### Histopathology

On day 6 post-BMT, colons and lungs from uninfected or *Hpb*-infected WT BALB/c, Il4Rα^−/−^, and RAG^−/−^ mice were fixed in 4% neutral buffered formalin and processed. Six-micrometer sections were cut and stained with H&E. Tissues were then analyzed for GVHD-related inflammation, and the severity of inflammation was scored in a blinded fashion. GVHD-related colitis was scored as previously described (25) as follows: none (score of 0), mild (1), moderate (2), severe without ulcer (3), and severe with ulcer (4). Crypt apoptosis was graded as none (score of 0), <2 crypts per 10 containing an apoptotic body (1), 2–5 crypts per 10 containing an apoptotic body (2), most (>5) crypts containing an apoptotic body (3), and most crypts containing more than one apoptotic body (4). Scores ranged from 0 to 8. GVHD-related lung inflammation was graded based on the presence of perivascular cuffing, vasculitis, peribronchiolar cuffing, and alveolar hemorrhage. Scores ranged from 0 to 4 (15, 22, 25–27).

### Statistical analysis

Differences in survival between groups were determined by Kaplan–Meier’s log-rank test. Differences in cell composition, cytokine content, and histopathological GVHD scores between infected and uninfected groups were determined using GraphPad Prism software, and significance was determined using an unpaired *t* test.

### Data availability

The data that support the findings of this study are available from the corresponding author upon reasonable request.

## Results

### The recipient Th2 pathway is essential for the survival of recipient T cells after BMT

**FIGURE 1.**
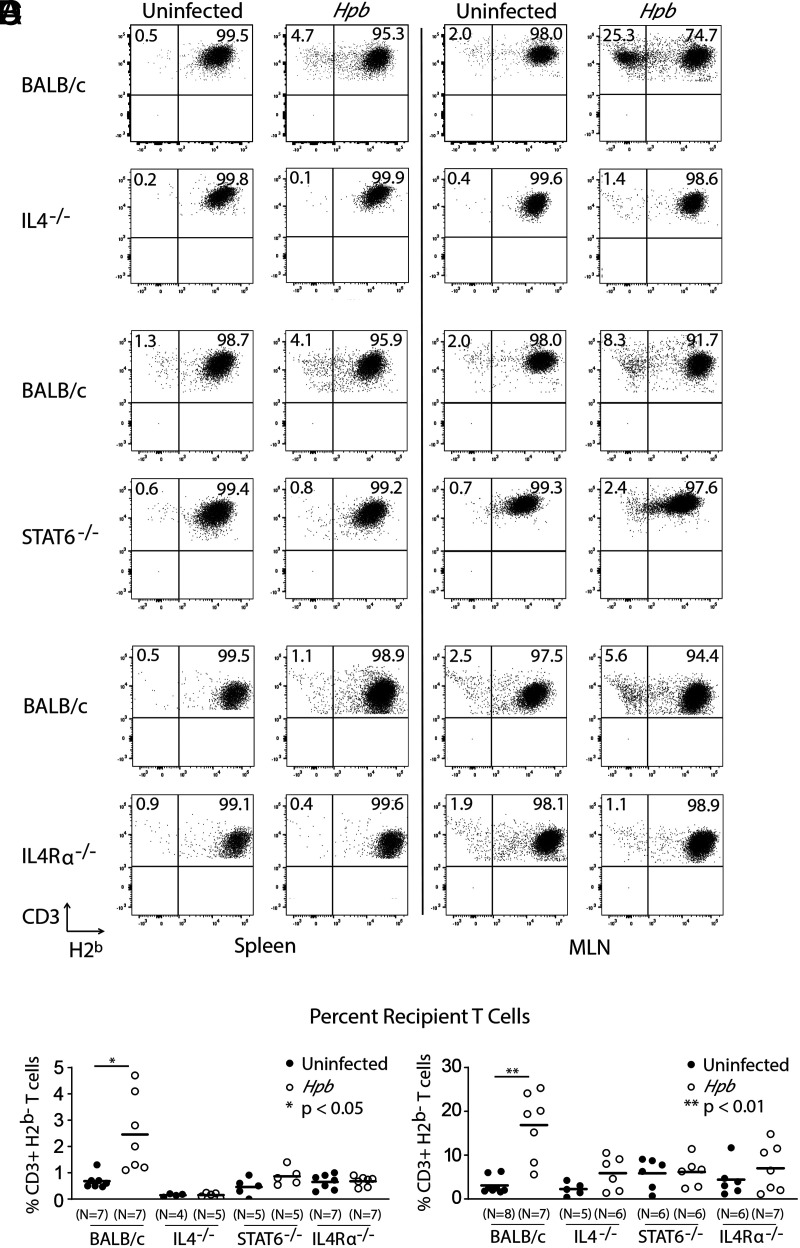
Recipient Th2 pathway is essential for recipient T cell survival after BMT. Splenocytes and MLN cells from uninfected and helminth-infected (*Hpb*) WT BALB/c, IL-4^−/−^, STAT6^−/−^ and IL-4Rα^−/−^ mice were isolated 6 d after BMT and analyzed for CD3 and H2^b^ (donor marker) expression by flow cytometry. (**A**–**C**) Representative example of dot plot analysis gated on CD3^+^ lymphocytes. Numbers in quadrants represent the percentage of events in each. Each parallel experiment (A, B, or C) in splenic T cell assessment contained one or two uninfected and one or two *Hpb*-infected WT BALB/c BMT recipient mice besides a group of uninfected and *Hpb*-infected Th2^−/−^ (IL-4^−/−^, A; STAT6^−/−^, B; or IL-4Rα^−/−^, C) BMT mice. Each parallel experiment (A, B, or C) in MLN T cell assessment contained one or two uninfected and 1 *Hpb*-infected WT BALB/c BMT recipient group for each group of uninfected and *Hpb*-infected Th2^−/−^ (IL-4^−/−^, A; STAT6^−/−^, B; or IL-4Rα^−/−^, C) BMT mice. Each experiment was repeated twice for splenic T cell quantitation in IL-4^−/−^, STAT6^−/−^, and IL-4Rα^−/−^ mice. Each experiment was repeated four times for MLN T cell quantitation in IL-4^−/−^, three times for STAT6^−/−^, and three times for the assessment of IL-4Rα^−/−^ mice. In each parallel experiment, WT donor C57BL/6 cells from the same donor were used to induce GVHD in uninfected and *Hpb*-infected Th2-sufficient or -deficient recipient pairs. (**D**) Quantitation of cumulative data from multiple experiments, where each dot (*N*) represents an independent experiment (an individual spleen [left] or group of pooled MLN [right] cells from two to three mice to obtain a sufficient cell number) and the bars represent the means for multiple independent experiments. For each BMT recipient strain, differences between uninfected and *Hpb*-infected groups were determined using an unpaired *t* test. *p < 0.05, **p < 0.01 between uninfected and *Hpb*-infected mice.

Helminthic regulation of GVHD requires the generation of Th2 cytokine IL-4 and signaling by the Th2-associated transcription factor STAT6 in recipient cells (14, 17). As helminthic regulation of GVHD is also associated with the survival of recipient CD3^+^ T cells after BMT (18), we investigated whether the recipient Th2 pathway plays a role in recipient T cell survival after helminth infection. We quantitated the expression of CD3, H2^b^ (donor cell), and H2^d^ (recipient cell) in splenic and MLN cell populations from uninfected and *Hpb*-infected Th2-sufficient BALB/c WT mice as well as several lines of Th2-impaired (IL-4^−/−^, STAT6^−/−^, IL-4Rα^−/−^) mice 6 d after BMT. The IL-4Rα-chain is a component of cellular Th2 cytokine complexes (IL-4 receptor and IL-13 receptor) that signal through phosphorylation of STAT6 (28). Initially, we performed staining for H2^b^ and H2^d^ besides CD3 in some samples. These pilot studies showed that the percentages of H2^b−^ T cells were comparable to the percentages of H2^d+^ T cells (and vice versa) in BALB/c WT, STAT6^−/−^ (Fig. 1, Supplemental Fig. 1), IL-4^−/−^ and IL-4Rα^−/−^ (data not shown) samples. We next tested a large cohort of BMT mice for CD3 and H2^b^ expression, discovering that in WT mice a significant portion of CD3^+^ cells did not stain for H2^b−^ following helminth infection but that almost all CD3^+^ cells from IL-4^−/−^, STAT6^−/−^, or IL-4Rα^−/−^ BMT recipients did (Fig. 1). Thus, helminth infection promotes survival of recipient T cells in the spleen and MLNs in WT but not IL-4^−/−^, STAT6^−/−^, or IL-4Rα^−/−^ BMT recipient mice when they receive WT C57BL/6 donor cells. Our findings suggest that both the production of Th2 cytokines by recipient cells as well as Th2 signaling resulting in STAT6 activity or signaling by receptor complexes (including IL-4Rα) are important for promoting recipient T cell survival (Fig. 1). These data represent solid evidence that the Th2 pathway of the recipient is essential for helminth-induced recipient T cell survival.

In another set of samples, we assessed the cell-intrinsic role of Th2 signaling in recipient T cell survival after BMT. We used mice with T cell–specific and conditional deficiency of the IL-4Rα-chain, generated by crossing T cell–specific iLck Cre transgenic (H2^d^) mice with a mouse strain that carries a targeted mutation of the IL-4Rα gene (IL-4Rα^flox/flox^) (H2^d^) (20). Helminth infection did not increase the percentage of recipient T cells in BMT recipient mice with T cell–specific Th2 deficiency (iLck Cre × IL-4Rα^flox/flox^; CKO [H2^d^]), which received WT donor cells from uninfected C57BL/6 (H2^b^) mice (Table I), although helminth infection increased the splenic recipient T cell percentage among total T cells in WT counterpart (non-Cre littermate control) BMT recipients (H2^d^) of C57BL/6 (H2^b^) donors. This group of studies suggests that survival of recipient T cells after BMT in helminth-infected mice involves activation of the cell-intrinsic Th2 pathway by intestinal helminth colonization. Thus, helminth-induced IL-4 production by recipient cells, which is critical to regulation of GVHD (14), has a direct effect on recipient T lymphocytes and promotes their survival.

**Table I.** Percentage of recipient T cells among all splenic T cells in BMT recipients of C57BL/6 donors

	CKO Mouse (Cre^+^)			Non-Cre Littermate Control (Cre^−^)		
	Uninfected (*n* = 3)	*Hpb* (*n* = 3)	*p* Value	Uninfected (*n* = 3)	*Hpb* (*n* = 3)	*p* Value
Recipient T cell % (mean ± SEM)	7.87 ± 3.90	4.03.0 ± 2.05	NS	5.23 ± 1.18	17.67 ± 1.52	<0.01

### Helminth-induced regulation of GVHD requires recipient cell expression of IL-4Rα

**FIGURE 2.**
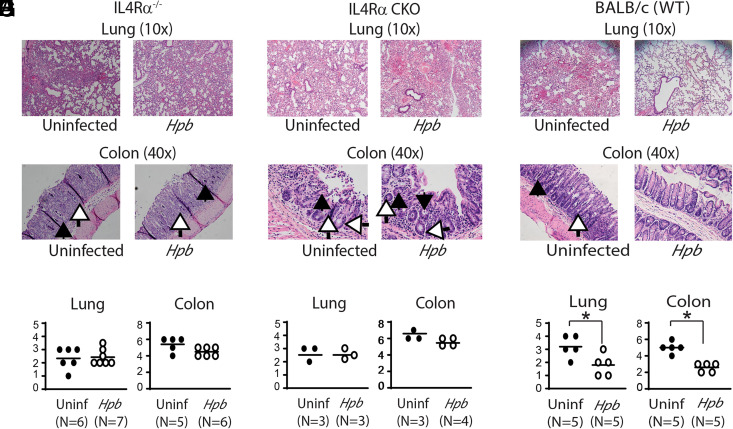
Helminths do not regulate GVHD-associated end-organ damage in IL-4Rα^−/−^ or T cell specific IL-4Rα–deficient BMT recipients. (**A**–**I**) Histopathological analysis of lung (original magnification, ×10 for A, D, and G) and colon (original magnification, ×40 for B, E, and H) from uninfected (Uninf) and *Hpb*-infected IL-4Rα^−/−^ (A–C) or T cell–specific IL-4Rα^−/−^ (conditional knockout [CKO]) (D–F) WT BALB/c or (G–I) BMT recipients, harvested 6 d after BMT. Arrows point to apoptotic crypt abscesses (black) and apoptotic bodies (white). Cumulative data are shown from two independent experiments and multiple samples of IL-4Rα^−/−^ (C), CKO (F), or WT BALB/c (I) BMT mice, where each symbol (dot) represents an individual sample (*N*) and the histopathological disease score for one mouse; bars represent means for multiple samples. Differences between groups were determined by unpaired *t* test. *p < 0.05 between uninfected and *Hpb*-infected mice.

Next, we investigated whether recipient T cell survival correlates with helminth-induced GVHD regulation, first by histopathological analysis and second by survival analysis of BMT mice. We showed before that helminths do not suppress GVHD in IL-4^−/−^ or STAT6^−/−^ BMT recipients (14, 17), where recipient T cells do not survive, as reported above (Fig. 1). In light of these findings, we explored whether helminthic regulation of GVHD is also impaired in IL-4Rα^−/−^ BMT recipients as well as CKO recipients. We found that *Hpb* infection did not suppress GVHD-associated end-organ damage in either Th2-deficient strain, as shown for the lung (Fig. 2A, 2D, 2E, 2H) and colon (Fig. 2B–D, 2F–H), although they did suppress GVHD-associated lung inflammation and colitis in their WT counterparts (Fig. 2I–L). Histopathological analysis of lungs from uninfected IL-4Rα^−/−^, T cell-specific IL-4Rα-deficient (CKO) and WT BALB/c BMT recipients revealed severe inflammation, alveolar hemorrhage, and loss of alveolar architecture (Fig. 2A, 2E, 2I). Similarly, colons from uninfected IL-4Rα^−/−^, CKO, or WT BALB/c BMT recipients showed severe inflammation characterized by the presence of mononuclear infiltrates, apoptotic bodies, and apoptotic crypt abscesses (Fig. 2B, 2C, 2F, 2G, 2J, 2K). Although colonization with *Hpb* larvae regulated inflammation in the lung and in colon of WT BALB/c BMT recipients (Fig. 2I–L), such treatment did not have a similar suppressive effect in IL-4Rα^−/−^ or CKO BMT recipients (Fig. 2A–H).

**FIGURE 3.**
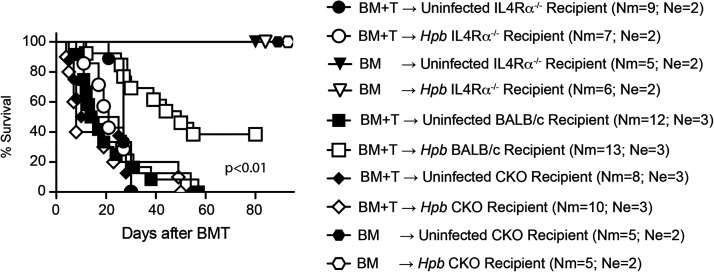
Helminth-induced promotion of survival of GVHD is dependent on IL-4Rα protein expression on recipient T cells. Kaplan–Meier survival curves are shown of *Hpb*-infected or uninfected IL-4Rα^−/−^, T cell-specific IL-4Rα^−/−^ (CKO), and WT BALB/c BMT mice, which received T cell–depleted bone marrow (TCD-BM) cells (BM) or TCD-BM plus total splenic T cells (BM+T) from C57BL/6 WT donors. Surviving mice were observed until day 80 after BMT. Cumulative data from multiple independent experiments (Ne indicates number of independent experiments for each uninfected and *Hpb*-infected BMT recipient strain pairs). In each experiment utilizing uninfected and helminth-infected recipient mice (WT BALB/c or gene-deficient [IL-4Rα^−/−^ or CKO]), same donor cells from C57BL/6 WT mice were used in adoptive transfer to uninfected and helminth-infected recipients of the same recipient colony (WT BALB/c, IL-4Rα^−/−^ or CKO). Nm indicates cumulative number of BMT mice in each group; *p* < 0.05 between *Hpb*-infected IL-4Rα^−/−^ mice injected with TCD-BM + T donor cells and *Hpb*-infected BALB/c WT recipients injected with TCD-BM + T donor cells; also, *p* < 0.05 between *Hpb*-infected CKO mice injected with TCD-BM + T donor cells and *Hpb*-infected WT recipients injected with TCD-BM + T donor cells.

In addition to failing to regulate histological GVHD in IL-4Rα^−/−^ or CKO mice undergoing BMT, helminth infection offered no survival advantage in the same circumstances (WT C57BL/6→IL-4Rα^−/−^ BMT or WT C57BL/6→CKO BMT), although it promoted the survival of some WT BALB/c recipients (Fig. 3), as was shown previously (14, 17, 18). Both uninfected and *Hpb*-infected IL-4Rα^−/−^ mice died of severe GVHD by day 30 after BMT (Fig. 3). In a similar way, uninfected and *Hpb-*infected mice with T cell–specific deficiency of the IL-4Rα-chain died of GVHD by day 54 after BMT. Similar to the IL-4Rα^−/−^ BMT groups, uninfected WT BALB/c BMT recipients also died of severe GVHD. In contrast, 5 of 13 (38%) of the *Hpb*-infected WT BALB/c recipients survived (Fig. 3). All mice in additional control groups (either uninfected or helminth-infected IL-4Rα^−/−^ or CKO BMT recipients transplanted with TCD-BM without splenic donor T lymphocytes from C57BL/6 WT mice) survived to the endpoint of 80 d (Fig. 3). Thus, helminths regulated GVHD in mouse strains, in which the recipient Th2 pathway is intact and not in strains, in which the recipient Th2 pathway is impaired. In the same framework where the recipient Th2 pathway or recipient T cell–specific Th2 signaling are impaired, recipient T cells did not survive during GVHD, and in BMT recipients in which recipient T cell number did not increase after helminth infection, helminths did not regulate GVHD. These results suggested that recipient T cells can be key players in Th2- and TGF-β–dependent regulation of GVHD.

### Helminth-induced survival of T cells after TBI is Th2-dependent

**FIGURE 4.**
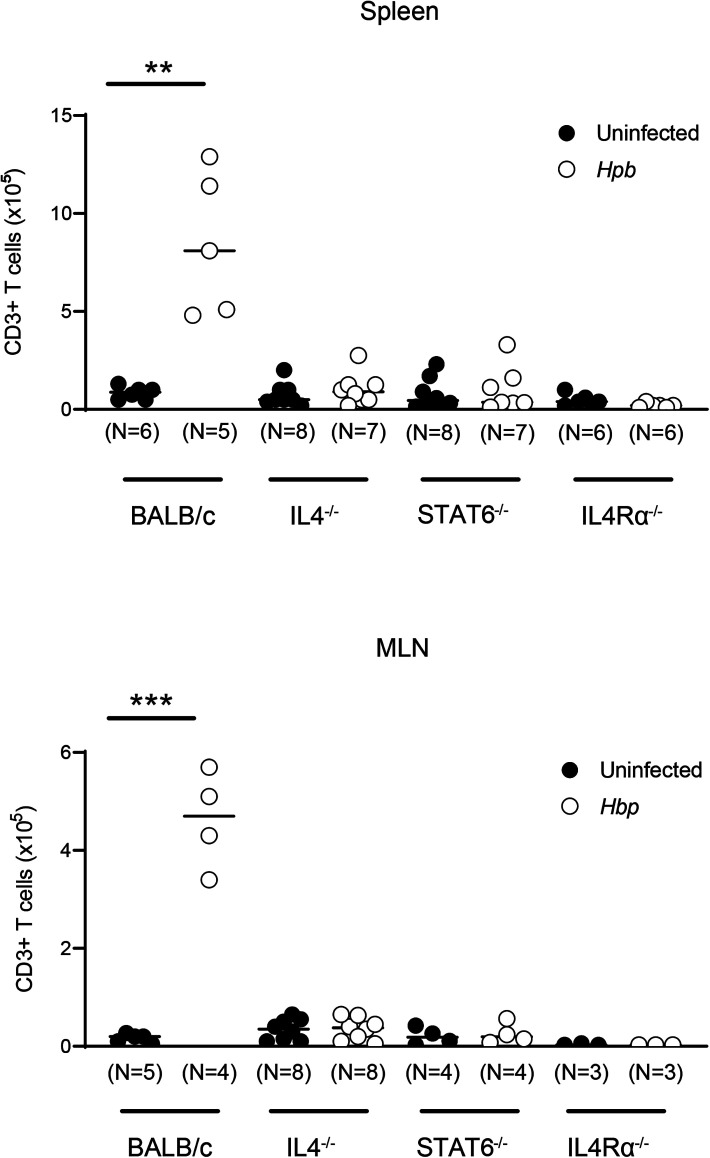
Helminths promote survival of recipient T cells following TBI and this is dependent on the Th2 pathway. Splenocytes (top) and MLN cells (bottom) were isolated from uninfected and *Hpb*-infected WT BALB/c, IL-4^−/−^, STAT6^−/−^, and IL-4Rα^−/−^ mice 6 d after TBI and stained for CD3. The total number of CD3^+^ T lymphocytes was calculated based on the total number of cells isolated and the percentage of CD3^+^ cells. Cumulative data from three independent experiments in spleen cells and MLN cells for each group (with an additional three independent experiments in MLN cells from only uninfected and *Hpb*-infected IL-4^−/−^ mice). *N* indicates the number of individual experiments in each group (each dot), where the number of cells from an individual spleen or pooled cells from MLN of a group of two to three mice constituted each dot;bars represent mean determinations from *N* individual experiments. **p < 0.001, ***p < 0.0001 between uninfected and *Hpb*-infected mice.

**FIGURE 5.**
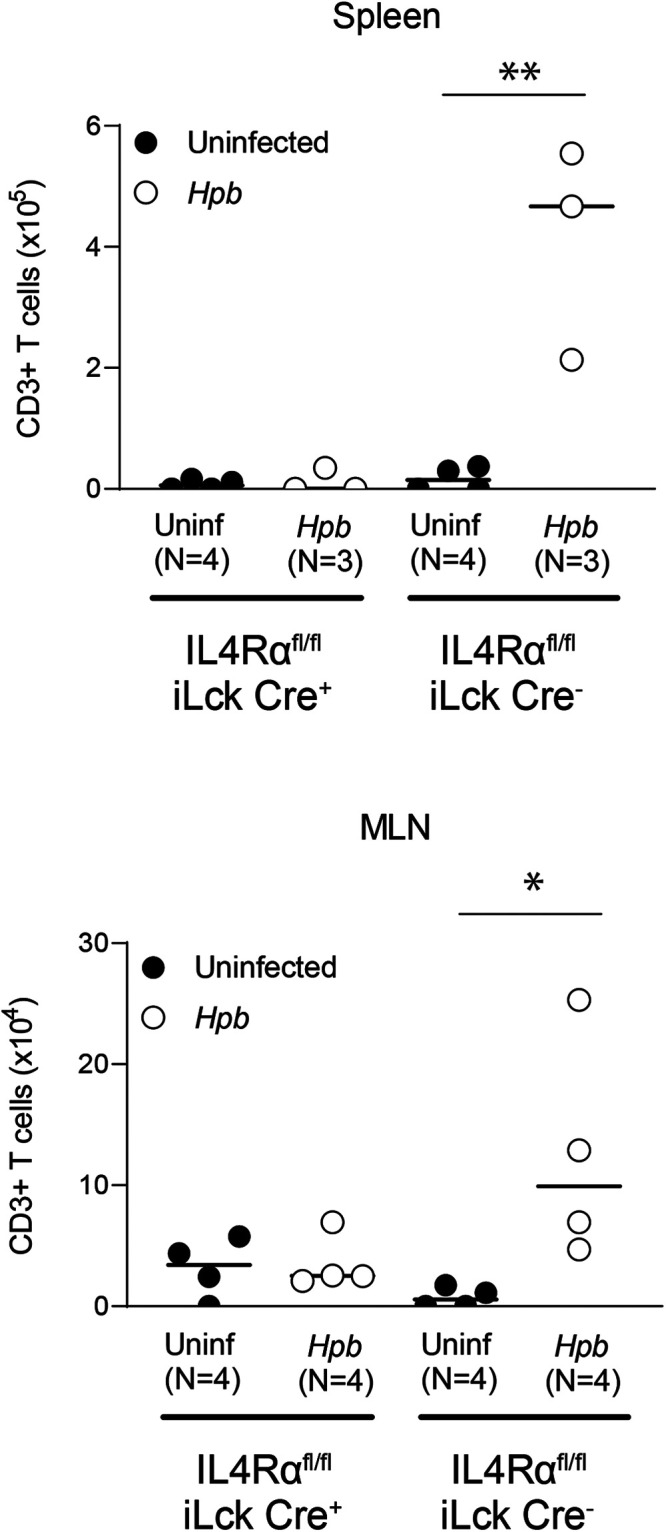
Cell-intrinsic IL-4Rα expression on recipient T cells is essential for cellular survival after TBI. Numbers of splenic (top) and MLN (bottom) cells positive for CD3 expression in uninfected and helminth-infected mice with a T cell–specific deficiency for IL-4Rα (IL-4Rα^flox/flox^ × iLck Cre^+^) (conditional knockout [CKO]) and their WT counterparts (non-Cre littermate controls; IL-4Rα^flox/flox^ × iLck Cre^−^) are shown. The total number of CD3^+^ T lymphocytes was calculated based on the total number of cells isolated and the percentage of CD3^+^ cells. Cumulative data from three independent experiments for spleen cells and four independent experiments for MLN cells. *N* indicates the number of individual experiments in each group (an individual spleen or pooled MLN cells from two to three mice); data bars represent mean determinations from *N* individual experiments. *p < 0.05, **p < 0.01 between uninfected and *Hpb*-infected mice.

To understand why the recipient T cells were not present in increased numbers after helminth infection in Th2-deficient BMT recipients, we first tested the possibility that recipient T cells may not survive the preparation with TBI before BMT. We assessed the survival pattern of T cells after irradiation in uninfected and *Hpb*-infected WT BALB/c, IL-4^−/−^, STAT6^−/−^, and IL-4Rα^−/−^ mice. In WT BALB/c mice, the numbers of both spleen- and MLN-derived CD3^+^ T cells that survived irradiation were higher after *Hpb* infection (Fig. 4). In contrast, in uninfected mice whose ability to upregulate Th2 signaling is impaired (IL-4^−/−^, STAT6^−/−^, or IL-4Rα^−/−^ genotype), these numbers remained low and did not rise above baseline levels after helminth infection (Fig. 4). The effect of this upregulation of Th2 pathway signaling on T cell resistance following preparation of the recipient mice with TBI was direct because helminth infection did not lead to a similar increase in the number of surviving T cells in CKO mice (Fig. 5), in agreement with recipient T cell counts after BMT in recipients with T cell–specific IL-4Rα deficiency (Table I). These findings suggest that helminthic stimulation of Th2 signaling has a direct effect on T cells and that this promotes the survival of recipient T cells after TBI. Upregulation of Th2 signaling by helminth infection might contribute to recipient T cell survival through a mechanism of radioresistance, in other words resistance to the preparation regimen prior to BMT. Furthermore, most surviving lymphocytes from non-Cre littermate controls of CKO mice were CD4 T cells (Table II), similar to our results in WT BALB/c mice (18). These results attest to a critical role of the cell-intrinsic Th2 pathway in mediating cellular resistance to radiation-induced cell death.

**Table II.** Percentage of CD4 T cells among surviving T lymphocytes

	CKO Mouse (Cre^+^)		Non-Cre Littermate Control (Cre^−^)	
	Uninfected	*Hpb*	Uninfected	*Hpb*
Spleen				
Nonirradiated*^a^* (mean ± SEM)	74.7 ± 2.1 (*n* = 3)	76.0 ± 1.9 (*n* = 3)	63.9 ± 1.6 (*n* = 3)	68.1 ± 0.9 (*n* = 3)
Irradiated*^b^* (mean ± SEM)	92.4 ± 0.7 (*n* = 4)	92.8 ± 0.9 (*n* = 3)	93.0 ± 1.1 (*n* = 4)	94.2 ± 0.5 (*n* = 3)
MLNs				
Nonirradiated*^a^* (mean ± SEM)	80.1 ± 2.8 (*n* = 3)	73.6 ± 2.5 (*n* = 3)	80.6 ± 0.7 (*n* = 3)	75.8 ± 0.6 (*n* = 3)
Irradiated*^b^* (mean ± SEM)	93.2 ± 0.3 (*n* = 4)	95.4 ± 2.0 (*n* = 4)	97.1 ± 0.7 (*n* = 4)	94.0 ± 0.5 (*n* = 4)

a Cell numbers were quantified in mice after 3 wk of helminth (*Hpb*) infection.

b Cell numbers were quantified in mice 6 d after TBI, which followed a 3-wk helminth (*Hpb*) infection.

### Th2-induced TGF-β is important for helminth-induced promotion of recipient T cell survival

**FIGURE 6.**
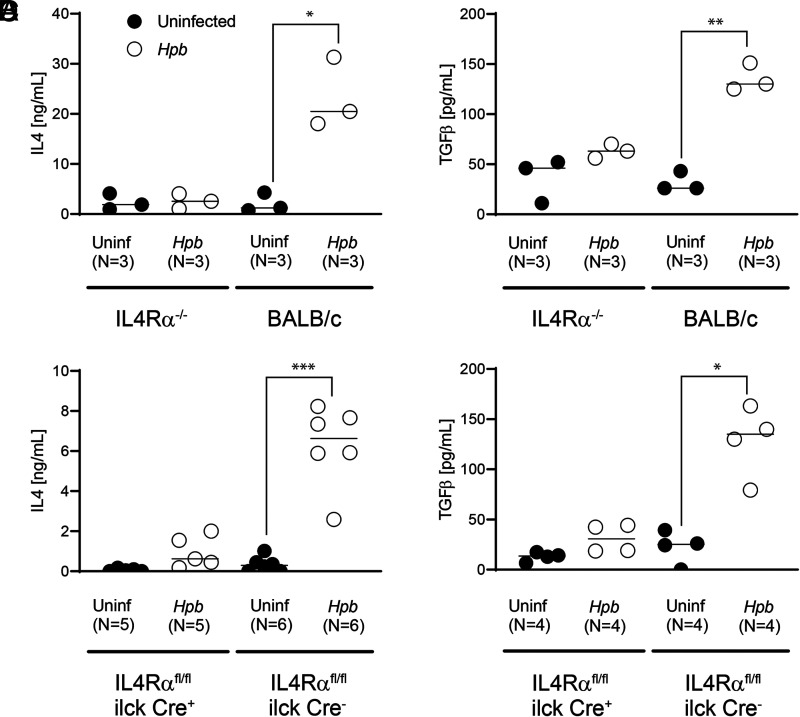
Helminthic induction of in vitro T cell–stimulated IL-4 and TGF-β production requires recipient cell IL-4Rα protein expression. (**A**–**D**) Concentration of IL-4 (A and C) and TGF-β (B and D) in supernatants from cultures of MLN cells from *Hpb*-infected and uninfected (Uninf) 8- to 9-wk-old male WT BALB/c or IL-4Rα^−/−^ mice (A and B) and T cell–specific IL-4Rα^−/−^ (CKO) mice (IL-4Rα^flox/flox^ iLck Cre^+^) and their WT counterparts (non-Cre littermate controls; IL-4Rα^flox/flox^ iLckCre^−^) (C and D), as measured by ELISA. Cells were stimulated in vitro with anti-CD3/28 for 48 h for IL-4 and for 72 h for TGF-β determination. Data show the mean (bar) from multiple independent experiments (scatter plots), where each dot (*N*) represents the mean value of a single independent experiment calculated from multiple repeats (three or more). Cumulative data from two independent experiments for IL-4Rα^−/−^ versus BALB/c comparisons and from four independent experiments for CKO versus non-Cre littermates control comparisons are displayed. Differences between groups were determined by an unpaired *t* test. *p < 0.01, **p < 0.001, ***p < 0.0001 for uninfected versus *Hpb*-infected mice.

We also tested the hypothesis that the cell-intrinsic Th2 pathway induces TGF-β production after helminth infection, as TGF-β appears critical to helminth-induced regulation of GVHD and helminths induce TGF-β production in a Th2-dependent manner (14, 17, 18). We first set out to determine the role that Th2 signaling plays in helminth-induced TGF-β production by T cells in uninfected and *Hpb*-infected IL-4Rα^−/−^ and CKO mice in the absence of BMT. Helminth infection did not augment the generation of IL-4 or TGF-β by T cells within MLNs in the absence of IL-4Rα expression (Fig. 6), narrowing down Th2-dependent TGF-β generation into a T cell–intrinsic regulatory circuit or developmental pathway.

**FIGURE 7.**
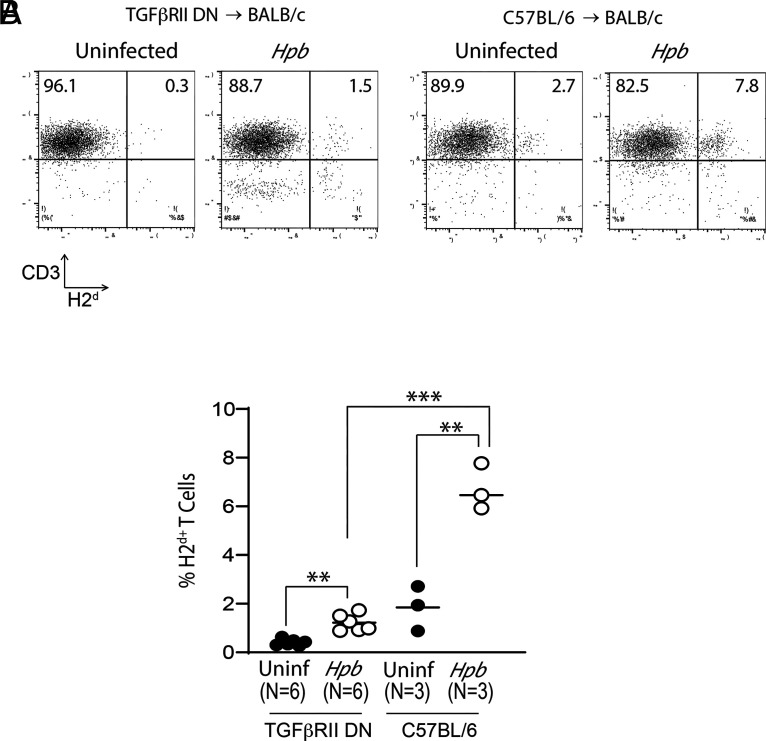
TGF-β signaling to donor T cells is essential for the survival of recipient T cells after BMT. CD3 and H2^d^ are shown in spleen cells from uninfected and *Hpb*-infected WT BALB/c BMT recipients of WT C57BL/6 (WT) or TGF-βRII DN splenic T cell donors, isolated 6 d after BMT. (**A**) Representative dot plot analysis from multiple experiments, with numbers in quadrants representing the percentage of events in each. (**B**) Cumulative data from three independent experiments, presented as scatter plots (each dot representing one spleen) and bar (mean). Each experiment used two uninfected BALB/c recipients of TGF-βRII DN donor T cells, two *Hpb*-infected BALB/c recipients of TGF-βRII DN donor T cells, one uninfected BALB/c recipient of WT C57BL/6 donor T cells, and one *Hpb*-infected BALB/c recipient of WT C57BL/6 donor T cells. Differences between groups were determined by an unpaired *t* test. **p < 0.01, ***p < 0.0001 between groups as indicated.

To determine whether TGF-β–mediated regulation of donor T cells contributes to recipient T cell survival after BMT, we investigated the distribution of donor and recipient T cells in WT BALB/c recipients where the donor T cells were deficient for TGF-β signaling because of overexpression of a truncated dominant-negative TGF-β receptor II protein (Cd4-TGFBR2 mice, in which CD8 T cells also express the truncated TGF-β receptor protein due to the absence of the CD8 silencer in the CD4 promoter construct) (29). Although the survival of recipient WT BALB/c T cells was promoted in a Th2-dependent manner in this context (Figs. 1, 4, and 5), recipient T lymphocytes constituted a sparse group among spleen cells; most of the T cell population in these animals had donor origin with impaired TGF-β signaling (Fig. 7). Thus, recipient T cells did not survive in BMT recipients of unregulated donor T cells with impaired TGF-β signaling. TGF-β is an essential regulator of acute GVHD (16, 18). Our findings now indicate that Th2 signaling can promote recipient T cell survival by promoting TGF-β production in a cell-intrinsic manner, thereby regulating donor T cell–mediated immune attack of GVHD and maintaining the survival of the recipient T cell population.

### The recipient T cell population is essential for the regulation of GVHD by helminths

**FIGURE 8.**
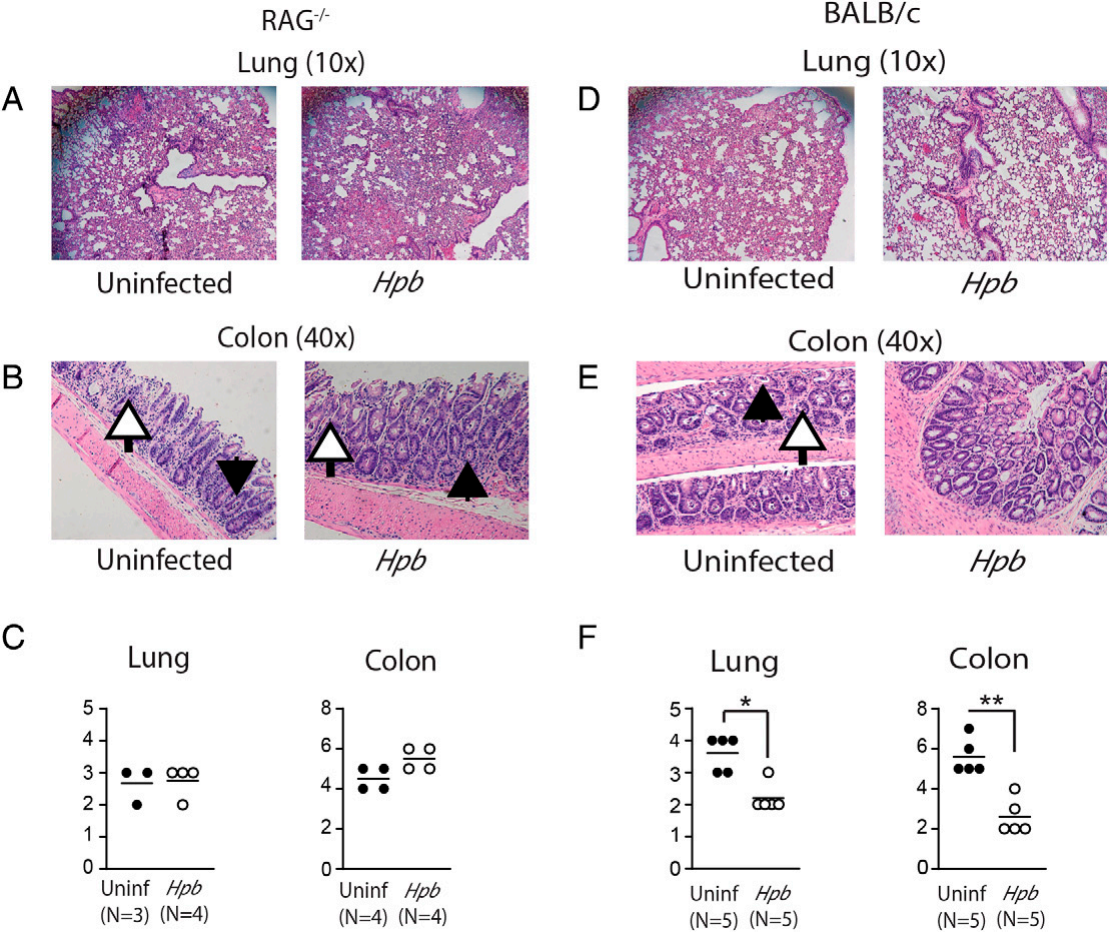
Helminths do not suppress GVHD-associated end-organ damage in RAG^−/−^ BMT recipients. (**A**–**F**) Histopathological analysis of lung (original magnification, ×10 for A and D) and colon (original magnification, ×40 for B and E) from uninfected (Uninf) and *Hpb*-infected RAG^−/−^ (A–C) or WT BALB/c (D–F) BMT recipients, harvested 6 d after BMT. Arrows point to apoptotic crypt abscesses (black) and apoptotic bodies (white). Cumulative data are from two independent experiments with multiple samples of RAG^−/−^ (C) or WT BALB/c (F) BMT mice, where each symbol (dot) represents an individual sample (*N*) and the histopathological disease score for one mouse; bars represent means for multiple samples. Differences between groups were determined by an unpaired *t* test. *p < 0.05, **p < 0.01 between uninfected and *Hpb*-infected mice.

Our data suggest that recipient T cells are part of a regulatory immune response that suppresses alloreactive donor T cells. However, it is also possible that recipient T cell survival is a consequence, rather than a cause, of GVHD suppression, as suggested by the absence of recipient T cells in helminth-infected WT BALB/c BMT mice, which received donor T cells deficient for TGF-β signaling (Fig. 7). A third possibility is that surviving recipient T cells can also aggravate GVHD (13, 19). To explore the biological significance of recipient T cell survival in helminth-induced regulation of GVHD, we employed a model of BMT involving helminth-infected and uninfected BALB/c RAG^−/−^ recipients (H2^d^) of WT C57BL/6 donors (H2^b^). RAG^−/−^ mice do not have peripheral B, T cells, invariant NKT (iNKT) cells but still preserve the NK cells. Given that peripheral B cells are absent during acute GVHD, as we also verified in helminth-infected BMT recipients (18) and as we showed before that recipient iNKT cells are redundant in helminth-induced regulation of GVHD (14), RAG^−/−^ mice constitute a reasonable choice as BMT recipients when investigating the role of recipient T cells in helminthic immune regulation of GVHD (10, 30, 31). Colonization of these mice with *Hpb* following BMT did not improve the end-organ damage, as judged by the presence of inflammatory infiltrates in both lung and colon (Fig. 8A–D), whereas the damage in WT BALB/c control BMT mice was significantly reduced (Fig. 8E–H). In addition, neither colonic inflammation nor the density of apoptotic crypt abscesses improved in RAG^−/−^ BMT recipients after *Hpb* infection (Fig. 8B–D). In contrast, the suppressive effect in WT animals was robust (Fig. 8E–H).

**FIGURE 9.**
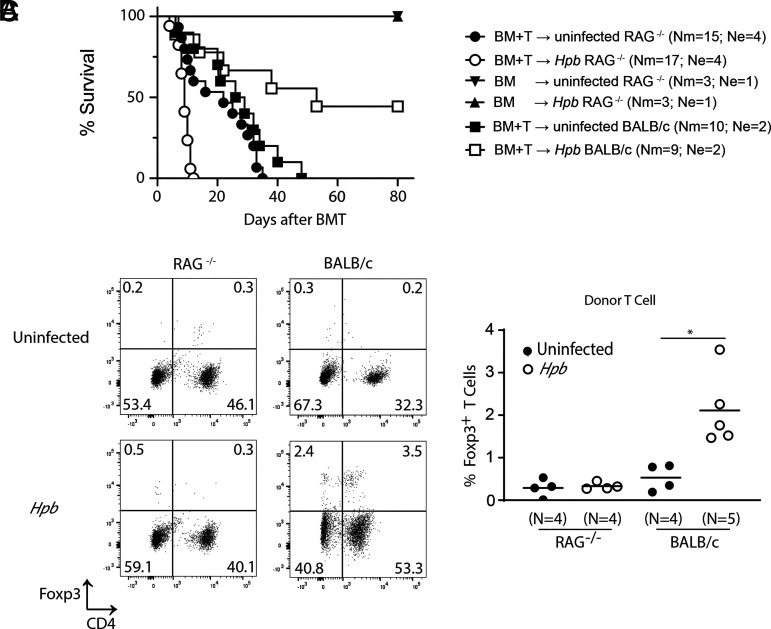
Helminth-induced expansion of donor Tregs and promotion of survival of GVHD is dependent on presence of recipient T cells. (**A**) Kaplan–Meier survival curves of *Hpb*-infected or uninfected RAG^−/−^ and WT BALB/c BMT mice, which received T cell–depleted bone marrow (TCD-BM) cells or TCD-BM plus total splenic T cells (BM+T) from C57BL/6 WT donors. Surviving mice were observed until day 80 after BMT. Cumulative data from multiple independent experiments (Ne indicates number of independent experiments for each uninfected and *Hpb*-infected BMT recipient strain pairs). In each experiment utilizing uninfected and helminth-infected recipient mice (WT BALB/c or RAG^−/−^), same donor cells from C57BL/6 WT mice were used in adoptive transfer to uninfected and helminth-infected recipients of the same recipient colony (WT BALB/c or RAG^−/−^). Nm indicates cumulative number of BMT mice in each group; *p* < 0.0001 between TCD-BM + T donor cells into *Hpb*-infected RAG^−/−^ and TCD-BM + T donor cells into *Hpb*-infected BALB/c WT recipients. (**B**) Foxp3 and CD4 expression in splenocytes from uninfected and *Hpb*-infected RAG^−/−^ and WT BALB/c BMT recipients of WT C57BL/6 splenic T cell and TCD-BM cell donors, isolated 6 d after BMT and stained for CD3, CD4, H2^b^, and Foxp3. Representative dot plot analysis of flow cytometry data gated on CD3^+^ and H2^b+^ lymphoid cells from multiple experiments, with numbers in quadrants representing the percentage of events in each. (**C**) Cumulative data from two independent experiments, with data displayed as scatter plots (each dot represents one spleen [*N*]) and bar (mean). Differences between groups were determined by an unpaired *t* test. *p < 0.05 between uninfected and *Hpb*-infected mice.

Consistent with the finding that helminth infection did not regulate end-organ damage in RAG^−/−^ BMT recipients, it also did not confer survival advantage to these animals. On the contrary, they exhibited accelerated mortality caused by GVHD, although some of the WT BALB/c BMT recipients (∼44%), in which recipient T cells persisted, survived (Fig. 9A). Control uninfected and helminth-infected RAG^−/−^ mice, which received donor TCD-BM cells but no splenic T cells, did not show mortality (Fig. 9A). These findings suggest that recipient T cells are essential to helminth-induced regulation of GVHD.

Foxp3^+^ regulatory T cells (Tregs) of donor origin represent one critical set of regulators of GVHD (32). Propagation of Foxp3^+^ Tregs in vivo has emerged as a means of enriching the donor T cell population for functional Tregs and thereby regulating GVHD (33). We previously showed that in vivo propagation of Tregs is dependent on Th2 cells and TGF-β (14, 17, 18). Likewise, in the current study, we found that the expansion of donor Tregs did not occur after helminth infection when recipient T cells conditioned to produce Th2 cytokines and TGF-β were not present (Fig. 9B, 9C). Taken together, our results showed that reprogrammed or conditioned recipient T cells, which produce IL-4 and TGF-β, are not only critical for the TGF-β–dependent expansion of donor Tregs (18) but also for the regulation of GVHD, filling an important gap in understanding our model, in which helminths induce T cell–intrinsic Th2 and TGF-β circuitries to suppress graft-versus-host reactivity.

## Discussion

Previous studies including our own observations have shown regulation of GVHD by conditioning of the BMT recipient’s immune system (12, 14, 17, 18, 34). Recipient cells can regulate GVHD by production of cytokines, such as the Th2 cytokine IL-4 (14, 15) that can be generated by T cells, mast cells, eosinophils, or basophils in significant quantities (35, 36). Our foregoing observations in STAT6^−/−^ BMT recipients also suggested that regulatory IL-4 production by recipients after helminth infection can act on recipient IL-4–responsive cell populations to regulate GVHD (17). Nonetheless, IL-4–producing and GVHD-regulating iNKT cells after nonmyeloablative preparation (15) appeared redundant (14), and mechanisms linking specific cell types of the recipient to Th2- and TGF-β–mediated regulation of GVHD has been unknown in our model of myeloablative BMT.

In this study, we demonstrate that IL-4 targets recipient T lymphocytes, constituting an IL-4 regulatory loop in helminth-induced regulation of GVHD. We also show that IL-4 targeting of recipient T lymphocytes promotes the survival of these cells after TBI and BMT, where the survival of recipient T cells appears to be critical in GVHD regulation. As most surviving recipient T lymphocytes after TBI are CD4 cells, our studies also provide a mechanistic connection between the Th2 pathway and CD4 T cell survival after irradiation. IL-4 targeting of recipient T lymphocytes further promotes TGF-β generation thereof, where TGF-β–dependent regulatory circuits promote recipient T cell survival (Fig. 7) and where TGF-β–dependent pathways are required for helminth colonization to regulate GVHD (18).

Investigating IL-4–mediated regulatory loops, we initially demonstrated Th2 dependence of recipient T cell survival after TBI in three different Th2-deficient strains, that is, in IL-4^−/−^, STAT6^−/−^, and IL-4Rα^−/−^ BMT recipient mice. Furthermore, that helminth infection did not promote T cell IL-4 production or, after TBI, T lymphocyte survival in a mouse model with T cell–specific deficiency of IL-4Rα (CKO mice) indicates that the Th2 cytokine IL-4 acts directly on T cells, attesting to the autocrine IL-4 and Th2 regulatory loop in alleviating GVHD. We found that most surviving T cells after TBI are CD4 T lymphocytes. Although radiation sensitivity of CD8 T cell subsets has been known for many years (37) and is actively investigated, the mechanisms of radiation sensitivity of CD4 T cells are not well characterized. A previous study showed preserved capacity of Th2 polarization and maintained expression of Th2 signaling intermediates of CD4 T cells after irradiation (38). In this context, our results establish a mechanistic connection between CD4 T cell–intrinsic Th2 signaling and cell survival after irradiation, which is required for helminth-induced creation of IL-4, the Th2 T regulatory loop, and regulation of GVHD in our myeloablative BMT model after TBI.

The second mechanism whereby the Th2 pathway promotes recipient T cell survival appears to involve stimulation of the TGF-β pathway, as we demonstrated previously and confirmed in the current study (18). In various diseases, TGF-β preserves intestinal and systemic immune homeostasis (39). Furthermore, data from several laboratories including ours suggest that TGF-β signaling is critical for helminth-induced immune suppression (40, 41). These reports have shown that TGF-β is required for helminth-induced suppression of colitis (41), multiple sclerosis (42), or GVHD (18), and also demonstrated that helminth products, instead of live helminths, promote immune regulation in a TGF-β–dependent manner, thereby contributing to regulation of reactive airway disease (40) in mice. Based on these studies, recent research has focused on the identification of helminthic TGF-β homologs in an attempt to develop these immune modulatory mediators for direct treatment of patients with immune diseases and eliminating the need to apply live helminths (43). Our findings demonstrate that recipient T cells do not survive in WT BMT recipients when T cells deficient for TGF-β T signaling are used as donors, even when recipient T cells survive TBI (18). This finding highlights the need to control donor T cells, which can kill recipient cells, in acute GVHD and shows that this requires TGF-β (16, 18). Because TGF-β production by recipient and donor T cells is triggered by helminth infection (18, 44), and this requires active Th2 signaling (14, 17), our studies imply that recipient T cells contribute to immune regulation and recipient T cell survival by either producing TGF-β or orchestrating Th2-driven TGF-β generation.

To characterize the role of recipient T cells and investigate helminthic regulation of GVHD in T cell–deficient recipients, we used a BMT and GVHD model utilizing C57BL/6 WT donors (H2^b^) and RAG^−/−^ recipients (H2^d^). RAG^−/−^ mice do not have T and B cells but still preserve the NK cells. As B cells are absent during GVHD (18), our studies of mortality in RAG^−/−^ recipient mice with an accelerated lethal GVHD after helminth infection shed light on the role of recipient T cells, supporting the notion that they play a critical role in suppressing GVHD after helminthic stimulation of the Th2 pathway. In combination with the fact that T cells are a robust source of Th2 cytokines after helminth infection (45, 46), our findings imply that recipient T lymphocytes, stimulated to produce IL-4 and TGF-β after helminth infection, are the critical drivers of Th2-dependent regulation of GVHD.

TGF-β contributes to GVHD suppression at multiple steps (16), including the expansion of Foxp3^+^ donor Tregs (14, 18). That donor Treg populations did not expand in RAG^−/−^ recipients indicates that recipient T cells may constitute a key cellular source of TGF-β for stimulating TGF-β–dependent expansion of donor Tregs. It is also possible that donor Tregs do not expand in the setting of more severe GVHD, in our case in severe GVHD of RAG^−/−^ BMT recipients. Studies of patients and various animal models of transplantation have shown that recipient- or donor-derived Foxp3^+^ Tregs are necessary for regulation of inflammation after syngeneic or allogeneic BMT/hematopoietic stem cell transplantation (10, 47, 48), with intestinal helminths or intestinal microbiota stimulating Tregs to regulate inflammation and alloreactivity (49, 50). Several clinical trials are either ongoing or are being designed to use or engineer Tregs capable of regulating inflammation and tissue damage (51). Tregs also appear to be essential to the suppression of anti-donor alloresponses, and they prevent the rejection of solid organs after combined transplantation (52). Thus, it will be interesting to see whether recipient or donor Tregs are required for the maintenance of recipient T cells and contribute to the regulation of GVHD in our model, in a comparable reciprocal way where recipient T cells are essential to expansion of donor Tregs and regulation of GVHD. It is also possible that a novel peripheral Th2 cell subset, which originates from Foxp3^+^ progenitors after *Hpb* infection, participates in Th2-driven regulation of GVHD because these novel Th2 cells, unlike conventional Th2 lymphocytes, regain the expression of Foxp3 after exposure to TGF-β (53).

Ongoing efforts attempt to promote recipient T cell (or other types of recipient hematopoietic cell) survival with their donor-derived counterparts to promote transplant tolerance in animal models and patients. For example, nonmyeloablative preparations, that is, preparation regimens that permit the engraftment of donor BM without eradicating recipient hematopoiesis, have led to successful survival of recipient T cells and the induction of immune tolerance after transplantation in mice (12, 34). In the same context, mixed chimerism has emerged as a preparation modality that permits survival of recipient and donor hematopoietic cells after transplantation to regulate GVHD or solid organ rejection (5, 54, 55). Mixed chimerism has been successfully applied to mice, large animals, and patients to promote immune tolerance (5) after transplantation. However, nonmyeloablative strategies have failed to reproducibly promote mixed chimerism and tolerance in nonhuman primates and patients (56, 57). Such approaches also have a disadvantage in clinical practice, in that they are associated with the recurrence of tumors after BMT is performed to eradicate hematological malignancies (54). Our studies indicate that the recipient T cell IL-4 and Th2 regulatory loop and TGF-β production promote recipient T cell survival, mimicking mixed chimerism, and regulate GVHD after myeloablative preparation (TBI) and also prevent tumor growth by preserving the graft-versus-tumor effect (18). Therefore, they deserve further attention in models of mixed chimerism after myeloablation. Indeed, recent small-scale studies have shown that inclusion of a Th2-inducing agent, such as G-CSF (58, 59), in even myeloablative preparation regimens appears promising for use to prevent GVHD in clinical practice. In other studies the use of G-CSF as part of the preparation regimen promoted the generation of TGF-β (16) and induced mixed chimerism (60).

A recent study has shown that IL-17–generating recipient T cells can drive GVHD (13). However, helminths regulate T cell–stimulated IL-17 generation in a Th2-dependent manner (61). According to our results, recipient T cells can be reprogrammed or immune conditioned to activate their Th2 pathway, generate TGF-β, and establish a regulatory autocrine IL-4 or Th2 loop after helminth infection. The same Th2-dependent pathways can also operate in mixed chimerism (34) where transient or durable recipient T cell survival can promote allotolerance after BMT (54, 55, 62). Helminth infection constitutes a safe approach with no severe side effects in clinical practice (63). With data accumulating that surviving recipient T cell populations can alter the course of GVHD by either aggravating or regulating it, further understanding the molecular mechanisms responsible for reprogramming or immune conditioning the recipient T cells could lead to discovery of more potent and less toxic protocols to promote specific immune tolerance in transplant patients.

## Supplementary Material

Supplemental Figures 1 (PDF)
