# Supplementary material for: After Bone Marrow Transplantation, the Cell-Intrinsic Th2 Pathway Promotes Recipient T Lymphocyte Survival and Regulates Graft-versus-Host Disease
Source: Immunohorizons. 2023 Jun 9;7(6):442–55. doi: 10.4049/immunohorizons.2300021 (PMC10580113; doi:10.4049/immunohorizons.2300021)
Supplement: Supplemental Figures 1 (PDF) [file IH_2300021_Supplemental_1.pdf]

Supplemental Information for Truscott et al. titled "After Bone Marrow Transplantation, Cell-Intrinsic T Helper-2 (Th2) Pathway Promotes Recipient T Lymphocyte Survival and Regulates the Graft-versus-Host Disease (GVHD)"

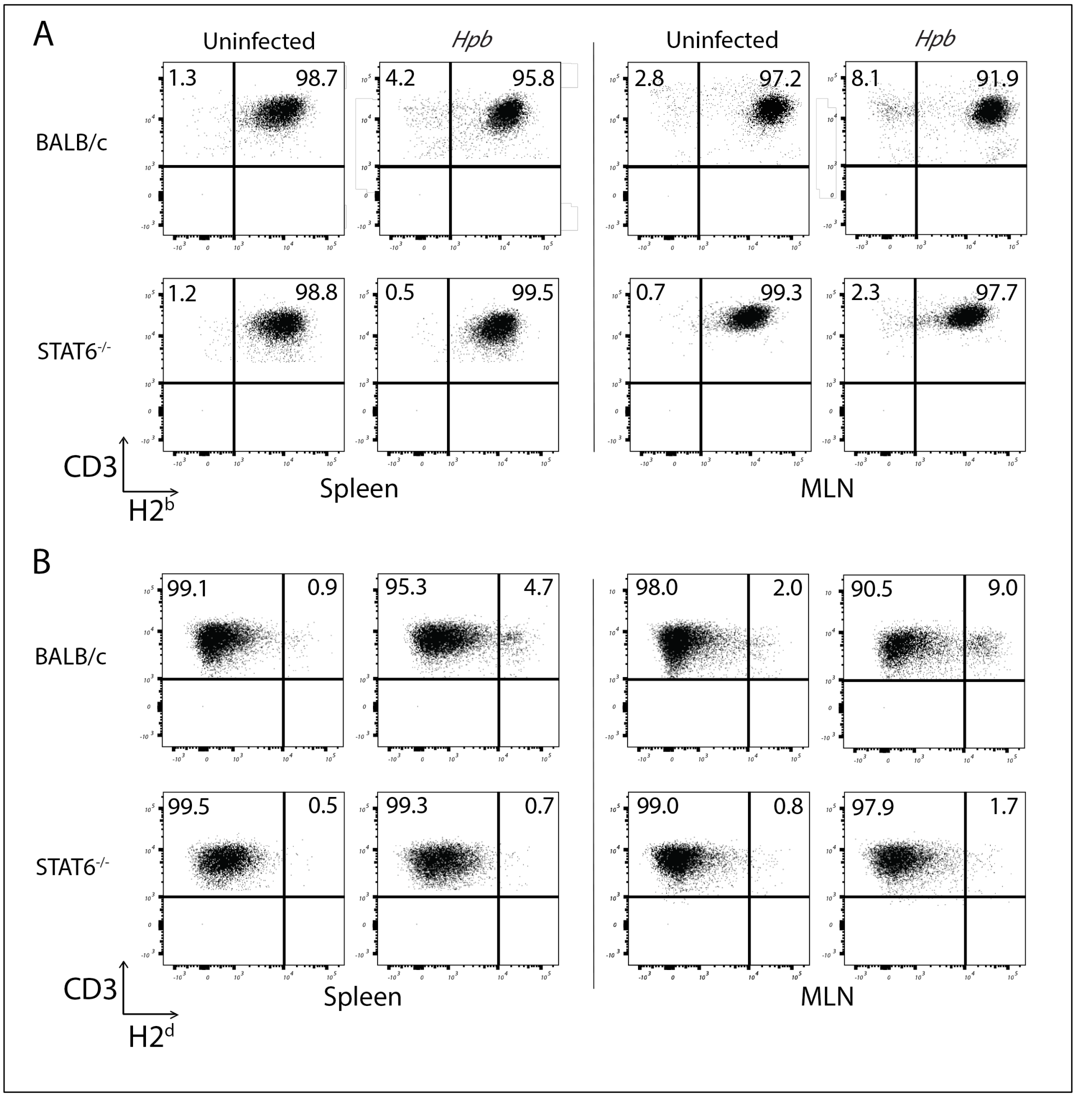

Supplemental Figure 1. Helminth-induced survival of recipient T cells after BMT requires STAT6 gene expression by recipient cells. Representative example from 2 independent experiments (for WT BALB/c vs STAT6$^{-/-}$ BMT recipients) for the expression of the donor marker H2^{b} (A) and recipient marker H2^{d} (B) in splenic (panels at left) and MLN (panels at right) CD3^{+} T cells isolated 6 days after BMT, from uninfected and *Hpb*-infected WT BALB/c and STAT6$^{-/-}$ mice, as indicated. The percentage of cells in each quadrant is shown.
